# miR-195-5p Regulates Tight Junctions Expression via Claudin-2 Downregulation in Ulcerative Colitis

**DOI:** 10.3390/biomedicines10040919

**Published:** 2022-04-16

**Authors:** Viviana Scalavino, Emanuele Piccinno, Antonio Lacalamita, Angela Tafaro, Raffaele Armentano, Gianluigi Giannelli, Grazia Serino

**Affiliations:** National Institute of Gastroenterology IRCCS “S. de Bellis”, Research Hospital, 70013 Castellana Grotte, BA, Italy; viviana.scalavino@irccsdebellis.it (V.S.); emanuele.piccinno@irccsdebellis.it (E.P.); antonio.lacalamita@irccsdebellis.it (A.L.); angela.tafaro@irccsdebellis.it (A.T.); raffaele.armentano@irccsdebellis.it (R.A.); gianluigi.giannelli@irccsdebellis.it (G.G.)

**Keywords:** miRNAs, IBD, tight junctions, claudins

## Abstract

Inflammatory bowel disease (IBD) is characterized by chronic intestinal inflammation associated with an increased intestinal permeability. Several studies have shown that microRNAs (miRNAs) are involved in the IBD pathogenesis. Here, we aimed to functionally characterize the role of miRNAs in the regulation of intestinal permeability and barrier function. We identified 18 dysregulated miRNAs in intestinal epithelial cells (IECs) from the ulcerative colitis (UC) mice model and control mice. Among them, down-regulated miR-195-5p targeted claudin-2 (CLDN2) and was involved in impaired barrier function. CLDN2 expression levels were increased in UC mice models and negatively correlated with miR-195-5p expression. We demonstrated that gain-of-function of miR-195-5p in colonic epithelial cell lines decreased the CLDN2 levels. This modulation, in turn, downregulated claudin-1 (CLDN1) expression at protein level but not that of occludin. Our data support a previously unreported role of miR-195-5p in intestinal tight junctions’ regulation and suggest a potential pharmacological target for new therapeutic approaches in IBD.

## 1. Introduction

Inflammatory bowel disease (IBD) includes distinct idiopathic disorders characterized by a chronic relapsing inflammatory state due to intestinal inflammation and epithelial injury. The two main disorders are Ulcerative Colitis (UC) and Crohn’s Disease (DC), which differ in terms of pathophysiology, the gastrointestinal area affected, and the depth of the inflammation in the intestinal wall [1]. The etiology of IBD is unknown, but several points of evidence suggest that IBD are multifactorial disorders in which genetic susceptibility, environmental factors, and an altered immune response interact, affecting intestinal homeostasis and barrier function [2].

Cell junctions between intestinal epithelial cells (IECs) regulate the paracellular permeability of the intestinal barrier. Cell junctions include tight junctions (TJ), adherens junctions (AJ), gap junctions, and desmosomes. TJs are multiprotein complexes located at the apical portion of lateral membranes of IECs which enclose the paracellular space. They act to maintain cellular polarity, regulate epithelial barrier function, and control paracellular permeability [3,4]. Most members of the claudins family contribute functionally to a paracellular tightening, other members form paracellular channels, and other claudins still have ambiguous functions. In the intestinal epithelium, claudins such as claudin-1, -3, -4, -5, -8 have a tightness function, while claudin-2 forms pore channels [5,6]. The restoration of TJ formations is one of the earliest cellular events in the intestinal wound-healing process [7].

During chronic inflammation, the epithelial barrier integrity is compromised, leading to an increased intestinal permeability. This could be associated with an alteration of claudins expression. Claudins dysregulation may contribute to an epithelial permeation disorder and multiple intestinal diseases, including IBD [8]. In fact, one common change is the increased expression of claudin- 2 (CLDN2) [9]. Overexpression of CLDN2 was shown to increase the number of pores that allow a paracellular flux of cations, contributing to the disease progression of IBD [10,11].

microRNAs (miRNAs) play a key role in regulating gene expression via post-transcriptional gene silencing. Over the past years, they have emerged as a main class of gene regulators in multiple biological pathways [12]. Several studies have also analyzed the correlation between miRNAs and many diseases, including IBD [13,14]. Investigations of miRNA profiles in IBD have improved our knowledge of their involvement in the IBD pathogenesis [15,16,17]. These studies have mainly been conducted on whole-tissue preparations deriving from IBD patients or IBD mouse models. This aspect is a limitation since intestinal tissue includes functionally and anatomically distinct layers, and in turn, each layer comprises multiple cell types. Therefore, understanding the role of specific miRNAs in a single cell type is essential to be able to interpret the biological function of these epigenetic regulators. Considering the pathophysiological relevance of intestinal permeability in IBD, the identification of miRNAs in IECs could provide new insight into the IBD pathogenesis and, therefore, treatment.

The aim of this study was firstly to identify the miRNAs expression profile of colonic IECs from an UC mice model and then to functionally characterize the role of miR-195-5p in the regulation of TJ protein expression and barrier integrity. This finding could suggest a strategy for the potential use of synthetic miRNA in IBD treatment.

## 2. Materials and Methods

### 2.1. Animal Experiments

The study was conducted in accordance with the ethical standards and according to national and international guidelines and had been approved by the authors’ institutional review board (Organism for Animal Wellbeing—OPBA).

All animal experiments were carried out in accordance with Directive 86/609 EEC enforced by Italian D.L. n. 26/2014 and approved by the Committee on the Ethics of Animal Experiments of the Ministero della Salute—Direzione Generale Sanità Animale (Authorization n. 337/2019-PR) and the official RBM veterinarian. Animals were sacrificed if in severe clinical conditions to avoid suffering.

### 2.2. Colonic Epithelial Cell Isolation

Four wild type mice and four Winnie mice were used for the IECs isolation. At 16 weeks, mice were sacrificed, and the colons were collected and digested for IECs isolation after mechanical and enzymatic digestion using the gentleMACS™ Dissociator and Lamina Propria Dissociation kit (Miltenyi Biotec, Bergisch Gladbach, Germany), respectively. IECs were selected using anti-CD326 (EpCAM) conjugated microbeads (Miltenyi Biotec, Bergisch Gladbach, Germany) for 15 min at 4 °C after CD45^+^ cells depletion. MACS separation was performed according to the manufacturer’s recommendation.

### 2.3. Total RNA Extraction and Microarray Expression Profile

Total RNA, including miRNAs, was extracted from isolated IECs and miRNAs were obtained using the miRNeasy mini kit (Qiagen, Hilden, Germany) following the manufacturer’s instructions. The RNA concentration and quality were determined with the NanoDrop ND-2000 Spectrophotometer (Thermo Fisher Scientific, Waltham, Massachusetts, MA, USA) and Agilent 2100 Bioanalyzer (Agilent Technologies, Palo Alto, CA, USA), respectively. Samples with RNA integrity ≥8.5 were used.

The miRNAs expression profile was analyzed using the Mouse SurePrint G3 miRNA microarray 8 × 60 K (Agilent Technologies, Palo Alto, CA, USA), based on Sanger miRBase release 21.0, according to the manufacturer’s instructions. Briefly, 100 ng of total RNA isolated from IECs were firstly dephosphorylated with calf intestine alkaline phosphatase treatment for 30 min at 37 °C before labeling. Samples were diluted with DMSO, denatured for 10 min at 100 °C, and labelled using pCp-Cy3 in T4 RNA ligation buffer. The labelled RNA was hybridized, washed, stained, and scanned with an Agilent microarray scanner (G2565BA, Agilent). Microarray data analysis was performed using Agilent Feature Extraction Software 12.1, leaving the default parameters.

Microarray data are available under accession number GSE183896 at the Gene Expression Omnibus (http://www.ncbi.nlm.nih.gov/geo/, accessed on 20 December 2021).

### 2.4. Cell Cultures

The human colon cell lines HT-29, Caco2, and T84 were obtained from ATCC (American Type Culture Collection, Manassas, VA, USA). All cell lines were cultured at 37 °C and 5% CO_2_. Caco2, HT-29 cell lines, were grown in Dulbecco’s Modified Eagle Medium (DMEM, Thermo Fisher Scientific) supplemented with 10% heat-inactivated Fetal Bovine Serum (FBS, Thermo Fisher Scientific), 10 mM HEPES (Sigma-Aldrich, St. Louis, MO, USA), 1 mM sodium pyruvate (Sigma-Aldrich), and 1% streptomycin/penicillin (Thermo Fisher Scientific). The T84 cell line was cultivated in Dulbecco’s Modified Eagle Medium: Nutrient Mixture F-12 (DMEM:F12, Thermo Fisher Scientific) supplemented with 10% FBS (Thermo Fisher Scientific, Waltham, MA, USA) and 1% streptomycin/penicillin (Thermo Fisher Scientific).

### 2.5. In Vitro Transfection

The cell lines were plated in 12 mm and 6.5 mm transwells (0.4 μm) (Corning, Corning, New York, NY, USA) until confluence. Following that, they were transfected with synthetic molecules of miR-195-5p mimic at concentrations of 30 nM and 50 nM (Life Technologies, Carlsbad, CA, USA) using TKO transfection reagent (Mirus Bio LLC, Madison, WI, USA), in accordance with the manufacturer’s instructions. Twenty-four and forty-eight hours after transfection, cell cultures were lysed for RNA isolation and protein extraction, respectively.

Mock controls, being cells transfected with transfection reagent without miRNA mimic, were included in all transfection experiments.

### 2.6. Real-Time PCR

Total RNA from IECs isolated from wild type mice and Winnie mice were reverse transcribed with the TaqMan Advanced miRNA cDNA Synthesis Kit (Thermo Fisher Scientific) following the manufacturer’s protocol. Real-time RT-PCR for the quantification of a group of selected miRNAs (miR-195a-5p, miR-497a-5p, miR-99a-5p, and miR-199a-3p plus an endogenous control) was performed with TaqMan Advanced miRNA assays and TaqFast Advanced Master mix (Thermo Fisher Scientific). Normalization was performed on the endogenous control miR-186-5p.

Total RNA was extracted from cell cultures using TRIzol reagent (Invitrogen, Carlsbad, CA, USA) according to the manufacturer’s protocol. RNA was reverse transcribed using the iScript Reverse Transcription Supermix (BioRad Laboratories, Hercules, CA, USA) following the manufacturer’s recommendations. qPCR amplification reactions were performed with SsoAdvanced Universal SYBR Green Supermix (BioRad Laboratories) using PrimePCR SYBR green Assay for claudin-2 (BioRad Laboratories) and QuantiTect Primer Assay for claudin-1 and Gapdh (Qiagen, Hilden, Germany). The relative expression of claudin 2 and claudin-1 was normalized using the Gapdh gene amplification as reference standard. Comparative real-time PCR was performed in triplicate, including no-template controls.

Real-time PCR amplification reactions were performed in 20 μL of final volume on a CFX96 System (Biorad Laboratories).

The relative expression for miRNAs and mRNA was calculated using the 2^−∆∆Ct^ method.

### 2.7. Western Blot

Total proteins were extracted from cell cultures using T-PER Tissue Protein Extraction Reagent (Thermo Fisher Scientific) supplemented with cocktail proteinase inhibitors (Sigma-Aldrich) and quantified by the Bradford protein assay (Biorad Laboratories). For each sample, equal amounts of proteins were loaded in 4–20% Mini-PROTEAN TGX Stain-Free Protein Gels (Biorad Laboratories) and then transferred to Trans-Blot Turbo Mini PVDF membranes (Biorad Laboratories). For protein detection, PVDF were incubated in iBind automated Western Systems (Thermo Fisher Scientific) following the manufacturer’s instructions. Immunoblot band densities were detected with an enhanced chemiluminescence method (Biorad Laboratories) using a Chemidoc System (Biorad Laboratories). Signal intensities were analyzed with Image lab Software (Biorad Laboratories) and quantified by ImageJ program. The protein expression values were normalized to housekeeping protein values.

For analysis, primary antibodies of mouse monoclonal claudin-1 (sc-166338, Santa Cruz Biotechnology, Inc., Heidelberg, Germany, dilution 1:1000), rabbit monoclonal claudin-2 (#48120, Cell Signaling, Technology, Danvers, MA, USA, dilution 1:1000), rabbit monoclonal Occludin (#91131, Cell Signaling, Technology, dilution 1:1000), and mouse mAb Tubulin (sc-166729, Santa Cruz Biotechnology, Inc., Dallas, TX, USA, dilution 1:1000) were used. Secondary antibodies used include Goat Anti-mouse IgG (H+L)-HRP conjugate (170-6516, Biorad Laboratories, dilution 1:500) and Goat Anti-rabbit IgG (H+L)-HRP conjugate (31466, Invitrogen, Carlsbad, CA, USA, dilution 1:2500).

### 2.8. Immunofluorescence

HT-29, Caco2, and T84 cells were seeded in 6.5 mm transwells (0.4 μm) (Corning) according to our experimental condition detailed in paragraph 2.4 and 2.5. The monolayers were washed three times with PBS and fixed with cold met-OH for 10 min at 4 °C. Subsequently, they were washed twice with PBS and permeabilized with Triton-X 0.1% in PBS for 5 min at room temperature, then samples were washed and blocked in PBS + BSA 3% for 1.5 h at room temperature. Following that, samples were incubated in primary antibody rabbit polyclonal claudin-2 (51-6100, Life Technologies, dilution 1:100) diluted in PBS + BSA 3% for 3 h. After washing with PBS, they were incubated with secondary antibody chicken anti-Rabbit IgG (H+L) Alexa Fluor 594 (A-21442, Invitrogen, dilution 1:400) diluted in PBS + BSA 3% for 1 h. ProLong Gold Antifade Mountant with DAPI (Thermo Fisher Scientific) was applied to each sample, mounted with a glass cover slip. Images were assessed using a fluorescence microscope (Eclipse Ti2, Nikon Inc., Melville, NY, USA) using filters for DAPI and RFP/TRITC.

### 2.9. Bioinformatic and Statistical Analysis

For microarray data analysis, the raw expression signals were log-transformed, normalized, and filtered according to the median corrected signal of all the miRNAs with an intensity >100 units (considered as expressed) and analyzed using Agilent GeneSpring GX 14.9 software. Probe sets were selected based on significant *p*-values and adjusted using the Benjamini–Hochberg FDR method. To determine differentially expressed miRNAs between the two groups, we applied a filter for FDR < 0.05 and a fold change (FC) of ±2. Hierarchical clustering and principal component analyses (PCA) were carried out with Genesis software (http://www.genesis-softwareonline.com/, accessed on 14 May 2021) using the average-linkage clustering method [18].

miRNA targets were predicted by means of miRbase 22.1 (Manchester, United Kingdom) [19], miRWalk 3.0 (Heidelberg, Germany) (http://mirwalk.umm.uni-heidelberg.de/, accessed on 21 May 2021) [20], TargetScan 7.1 (Cambridge, Massachusetts) (http://www.targetscan.org/vert_71/, accessed on 21 May 2021) [21], TarBase v.8 (Thessaly, Greek) (http://carolina.imis.athena-innovation.gr/diana_tools/web/index.php?r=tarbasev8/index, accessed on 21 May 2021) [22], and miRDB (St. Louis, Missouri) (http://www.mirdb.org/, accessed on 21 May 2021) [23] algorithms. Potential targets were chosen by overlapping results from the five algorithms and selecting targets of genes predicted by at least two of the algorithms. To assess biologic relationships among genes controlled by dysregulated miRNAs, we used miRSystem ver. 20160513 (Taipei, Taiwan) [24] and miRPath v.3 (Thessaly, Greek) [25] software.

Microarray GEO datasets GSE133059, GSE133060, GSE128682, and GSE87466 were used to evaluate the expression of miR-195-5p, CLDN1, CLDN2, and occludin. Differentially expressed genes or miRNAs were screened out with the threshold of |log2 FC| > 0.58 and FDR < 0.05 through several Bioconductor packages of framework R (Boston, Massachusetts) (https://www.r-project.org/, accessed on 06 September 2021). We used “limma” (Boston, Massachusetts) (https://bioconductor.org/packages/release/bioc/html/limma.html, accessed on 14 September 2021) or DESeq2 (Boston, Massachusetts) (https://bioconductor.org/packages/release/bioc/html/DESeq2.html, accessed on 14 September 2021) depending on the origin of the data (microarray or sequencing, respectively).

Statistical analysis was performed using GraphPad Prism software. Statistical significance of data deriving from different conditions was evaluated with two-tailed Student’s *t* test. A Pearson correlation test was applied to study continuous variables. All values are expressed as the mean ± SEM of data obtained from at least three independent experiments. Results were considered statistically significant at *p* <  0.05.

## 3. Results

### 3.1. miRNA Expression Profile of IECs from UC Mice Models

To identify the miRNAs that differentiate the colonic IECs of UC mice models (Winnie mice) and wild-type mice, we performed a miRNA expression profiling by microarray.

**Figure 1:**
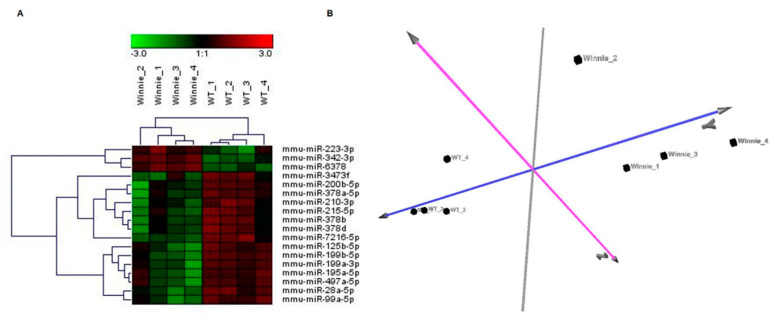
miRNA expression profile of colonic epithelial cells from Winnie and wild-type mice. (**A**) Hierarchical clustering using the 18 differentially expressed miRNAs (FDR < 0.05 and fold change threshold >2) discriminating colonic epithelial cells from Winnie mice compared to wild-type mice. Two principal clusters were identified on the basis of differential miRNA expression. (**B**) PCA based on the expression of differentially expressed miRNA in all samples. PCA showed evident clustering and confirmed the separation of Winnie and wild-type mice.

Of approximately 1881 miRNAs on the microarray, 460 miRNAs were expressed in colonic IECs. Differential expression analysis (FDR ≤ 0.05 and the fold change threshold ≥2) between 4 Winnie mice and 4 wild-type mice revealed 18 dysregulated miRNAs (Table S1). Hierarchical clustering analysis generated a tree that showed two clearly separate groups (Figure 1A). This separation was also confirmed by the correlations among miRNA expression patterns obtained using PCA (Figure 1B).

### 3.2. In-Silico Prediction of miRNA Targets

To determine the molecular mechanisms in which the 18 identified miRNAs are involved, we performed bioinformatic analysis to predict their target genes. Based on the results of the bioinformatic analysis, we found that one of the putative targets of miR-195a-5p was CLDN2, a gene that encodes a TJ protein expressed in colonic epithelium (Table S2).

Pathway analysis of dysregulated miRNAs revealed a rank-ordered list in which the top pathways were Focal Adhesion, MAPK signalling, Wnt signalling, Adherens junction, Gap junction, and Tight junction (Table S3). Of note, within these pathways, multiple miRNAs putatively regulated the same target genes, and one single miRNA could target several genes.

### 3.3. miRNA Expression Validation

**Figure 2:**
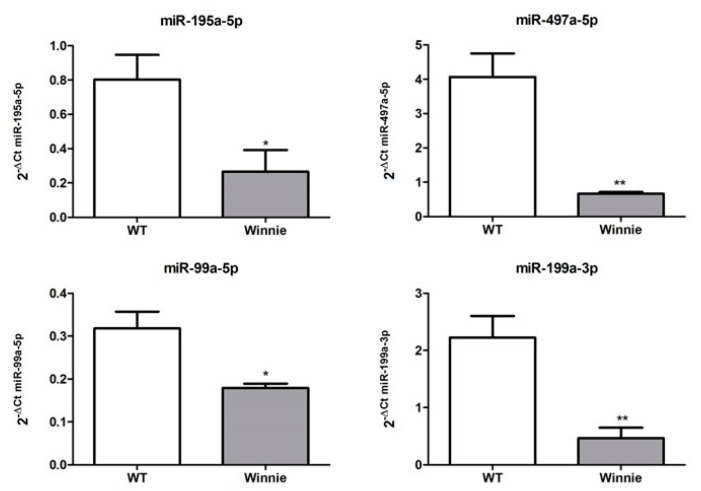
Validation of differentially expressed miRNAs. Expression levels of miR-195a-5p, miR-497a-5p, miR-99a-5p, and miR-199a-3p on miRNAs isolated from colonic IECs. miRNAs expression levels were quantified using qRT-PCR and the relative expression was normalized to the expression of endogenous control miR-186-5p. All tested miRNAs showed a significant change in expression, confirming microarray data. * *p* < 0.05; ** *p* < 0.01.

To validate the microarray results, we performed quantitative real-time PCR for miR-195a-5p, miR-497a-5p, miR-99a-5p, and miR-199a-3p on miRNAs isolated from colonic IECs. All tested miRNAs showed a significantly changed expression in Winnie mice compared to wild-type mice, confirming the microarray data (Figure 2).

### 3.4. miR-195a-5p as a Regulator of CLDN2 Expression

The increased expression of CLDN2 in IBD is widely recognized [10,26], but the basis for its increase is still unknown.

**Figure 3:**
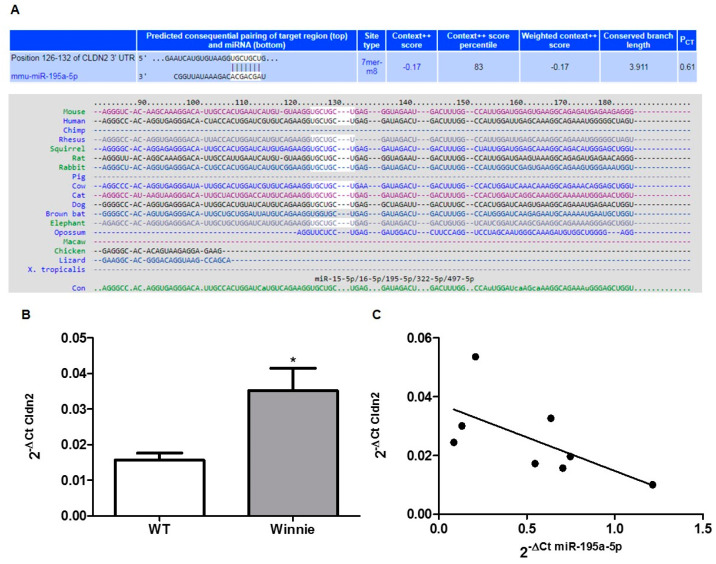
miR-195a-5p targets CLDN2. (**A**) Sequence alignment of the miR-195a-5p base-pairing sites in the 3’-UTR of Cldn2 mRNA showing that the regions complementary to miR-195a-5p are highly conserved among mouse, human, and chimp. The “seed” sequences of miR-195a-5p complementary to Cldn2 are shown in white. (**B**) Cldn2 mRNA expression levels evaluated in the same set of RNA samples used in the microarray validation. Cldn2 levels were significantly higher in Winnie mice compared to wild-type mice. Cldn2 expression levels were normalized on the housekeeping gene Gapdh. The histograms represent the mean ± SEM. * *p* = 0.03. (C) Linear correlation between the expression of Cldn2 and the expression of miR-195a-5p. Cldn2 mRNA levels inversely correlated with miR-195a-5p expression levels. Black points represent correlation between mRNA and miRNA expression for each sample (r = −0.64; *p* = 0.04).

The in-silico analysis showed that miR-195a-5p could regulate Cldn2 expression; in fact, sequence alignment of murine miR-195a-5p with 3’untranslated region (UTR) Cldn2 identified a binding site that is well conserved among different species (Figure 3A). The same binding site was observed in the corresponding 3’UTR human CLDN2 human gene.

Next, we evaluated the Cldn2 mRNA expression levels in the same set of RNA samples used in the microarray validation. We found that Cldn2 levels were significantly higher in Winnie mice compared to wild-type mice (*p* = 0.03; Figure 3B). Moreover, to study whether the upregulation of Cldn2 was attributable to lower levels of miR-195a-5p, we tested the correlation between miR-195a-5p and Cldn2 mRNA. A negative correlation was observed (r = −0.64; *p* = 0.04; Figure 3C).

### 3.5. miR-195-5p Regulates Cldn2 mRNA

**Figure 4:**
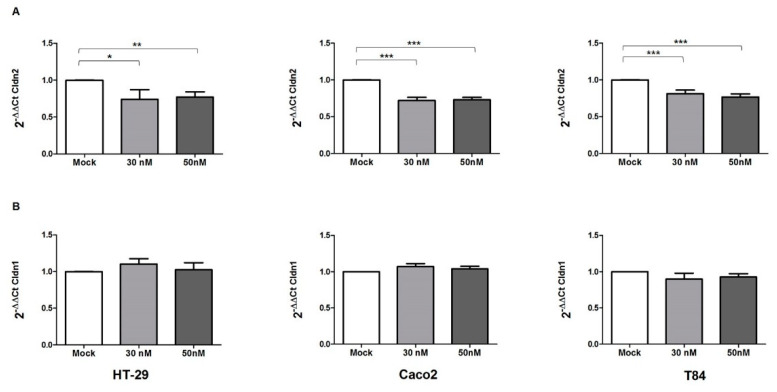
miR-195-5p regulates Cldn2 mRNA expression. (A) Cldn2 mRNA expression was analyzed by Real-Time PCR after transfection with miR-195-5p mimic in HT-29, Caco2, and T84 human cell lines. The increased intracellular amount of miR-195-5p (at 30 nM and 50 nM concentrations) led to a significant decrease of Cldn2 in all cell lines. (B) Cldn1 mRNA expression was analyzed by Real-Time PCR after transfection with miR-195-5p mimic in HT-29, Caco2, and T84 human cell lines. The levels of Cldn1 remained similar to mock-control in all cell lines. Expression data were normalized to the housekeeping gene Gapdh. Data are representative of four independent experiments (mean ± SEM). * *p* < 0.01, ** *p* < 0.001, *** *p* < 0.0001.

In order to validate the results of the bioinformatic analysis, we investigated the effect of miR-195-5p upregulation on the expression of the candidate gene Cldn2. We carried out in vitro transient transfection with molecules of miR-195-5p mimic in HT-29, Caco2 and T84 cell lines. After transfection, the levels of mRNA of Cldn2 were significantly decreased in all three cell lines of miR-195-5p mimic at concentrations of 30 nM and 50 nM (*p* < 0.01; Figure 4A). Moreover, we also evaluated whether the increase of miR-195-5p influenced the expression of Cldn1, a TJ component that is widely expressed in the intestine and has an important role in the integrity of the intestinal epithelium. After transfection, we found that the levels of Cldn1 remained similar to mock-control in all cell lines (*p* > 0.05; Figure 4B).

### 3.6. miR-195-5p Regulates CLDN2 at Protein Level

**Figure 5:**
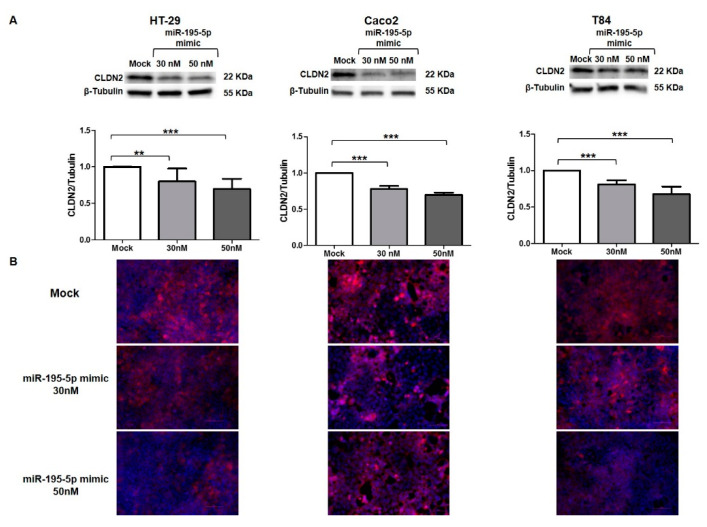
Regulation of CLDN2 protein expression by miR-195-5p in colonic epithelial cell lines. (**A**) Western blot analysis of CLDN2 protein expression in HT-29, Caco2, and T84 cell lines after miR-195-5p mimic transfection. A significant reduction of CLDN2 expression was detected in all cell lines. Raw data of the independent experiments of Western blot were reported in Appendix A. (**B**) Immunofluorescence staining of CLDN2 in HT-29, Caco2 and T84 cell cultures after miR-195-5p mimic transfection. In accordance with Western blot results, after transfection, the expression of CLDN2 decreased compared with mock-control. Single channel images of DAPI and CLDN2 staining before merge were reported in Appendix B. Data of WB were obtained by dividing the normalized transfected sample values by the normalized control sample values. β-Tubulin was used as housekeeping protein to normalize the data. Data are representative of four independent experiments. The histograms correspond to mean ± SEM. Scale bar = 50 µm ** *p* < 0.01; *** *p* < 0.001.

We used the same strategy to functionally enhance the mature form of miR-195-5p in HT-29, Caco2, and T84, with the aim of probing whether CLDN2 protein expression was also controlled by miR-195-5p. In accordance with the results obtained at the RNA level, Western blot analyses showed that CLDN2 protein expression decreased after miR-195-5p mimic transfection in all cell lines (*p* < 0.05; Figure 5A).

Moreover, we investigated the localization of CLDN2 in HT-29, Caco2, and T84 after miR-195-5p mimic transfection. As shown by immunofluorescence staining, in monolayer cell cultures CLDN2 was localized at the plasma membrane in all cell lines. In accordance with Western blot results, after the transient transfection with miR-195-5p mimic, the expression of CLDN2 decreased as compared with the mock-control (Figure 5B).

### 3.7. miR-195-5p Influences CLDN1 Expression but Not OCCLUDIN

**Figure 6:**
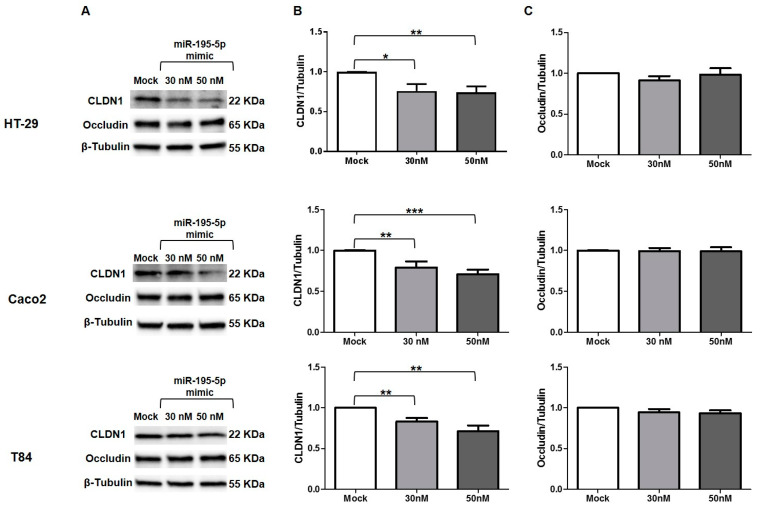
CLDN1 and Occludin protein expression after miR-195-5p mimic transfection. (**A**) Representative blots of CLDN1 and occludin protein expression in three IECs cell lines, HT-29, Caco2, and T84 after transfection. Western blot quantitative analysis demonstrated inhibition of CLDN1 (**B**) but not occludin (**C**) in all cell lines, after miR-195-5p mimic transfection at 30 and 50 nM. Mock represents cells which have undergone transfection without miRNA mimic. Raw data of the independent experiments of Western blot were reported in Appendix A. Data were obtained by dividing the normalized transfected sample values to normalized mock-control sample values. β-Tubulin was used as housekeeping protein to normalize the data. Data are representative of four independent experiments. The histograms correspond to mean ± SEM. * *p* < 0.05; ** *p* < 0.01, *** p < 0.001.

We also investigated the expression of CLDN1 at protein level, which was found to be significantly reduced (*p* < 0.05; Figure 6A,B). The discrepancy between mRNA and protein expression of CLDN1 after transfection could be due to an indirect link among miR-195-5p and CLDN1. The reduction of CLDN1 expression was probably the consequence of CLDN2 downregulation.

Another protein that plays a crucial role in the TJ structure and permeability of intestinal epithelial is occludin. This protein is not individually involved in TJ formation, but its recruitment requires the co-expression of claudins such as CLDN1 [27]. For these reasons, we also evaluated the protein expression of occludin after miR-195-5p transfection in all cell lines. We found that occludin expression after transfection was almost similar to mock conditions in all three cell lines (Figure 6A,C).

### 3.8. miR-195-5p and TJ Expression in UC Patients

To assess the potential clinical relevance of our findings, we analyzed four gene expression datasets from GEO (GSE133059, GSE133060, GSE12868221, and GSE8746622). These datasets included miRNA or mRNA expression profiles of patients with UC.

For gene expression analysis, we combined three datasets, GSE133060, GSE128682, and GSE87466. For the miRNA expression profile, we examined one dataset—GSE133059.

**Figure 7:**
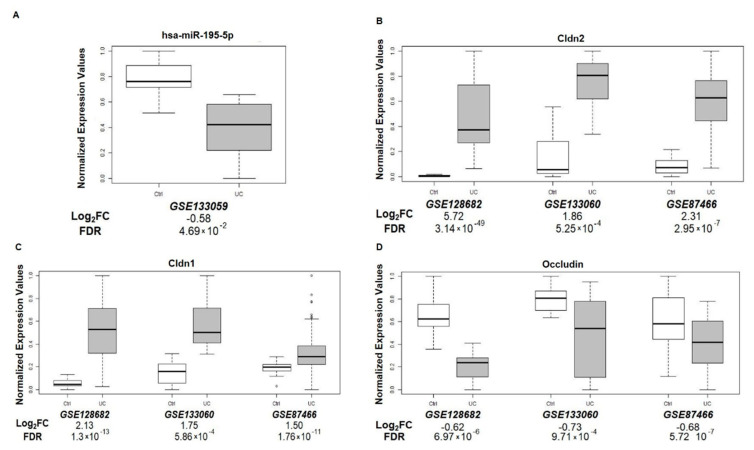
miR-195-5p, Cldn2, Cldn1, and Occludin expression in UC patients. Analysis of colonic tissue from UC and controls downloaded from the GEO database (GSE133059, GSE133060, GSE128682, and GSE87466). Mean expression data of miR-195-5p (**A**), Cldn2 (**B**), Cldn1 (**C**), and Occludin (**D**) were expressed as normalized expression values. LogFC value and FDR were reported for each dataset and each signal.

We found that miR-195-5p was significantly downregulated in UC patients compared to the control group (Figure 7A). Moreover, expression levels of CLDN1 and CLDN2 were significantly higher in UC patients compared to controls (Figure 7B,C) and occludin mRNA levels were reduced in UC patients (Figure 7D).

## 4. Discussion

Cell junctions are one of the physical barriers that can inhibit intestinal mucosa invasion by pathogenic and commensal microorganisms [28]. In the cell monolayer, TJs closely regulate paracellular transport and have a key role in the maintenance of intestinal permeability and barrier function [29,30]. In the IBD pathogenesis, the trigger event that culminates in intestinal inflammation is the increase of intestinal permeability due to dysregulation of proteins involved in cell junctions [4,10].

The implication of miRNAs in the IBD pathogenesis has gained broad interest over the years. miRNAs are implicated in the activation and maturation of immune cells and in the regulation of the immune system response [31,32,33,34,35]. Moreover, miRNAs also have a relevant role in IECs functions and may be implicated in enhancing the barrier function in the intestinal epithelium. For example, Dai and co-workers have shown that PepT1 is overexpressed in UC patients and its expression is modulated by the miR-193a-3p via NF-kB pathway [36]. Zhang et al. have identified overexpressed miR-21 in an in vitro model treated with TNF-α, highlighting its key role in the control of intestinal epithelial permeability [37]. In another work, Tian and colleagues found that MIR31 is increased in patients affected by IBD and, through the Wnt and Hippo pathways, is able to reduce inflammation and epithelial regeneration [38].

In this study, the IECs global miRNA expression profile of UC and wild-type mice identified 18 differentially expressed miRNAs. One of these, miR-195-5p, resulted in downregulation and caught our attention, since among their putative target genes there was CLDN2, a gene known to be upregulated in IBD [4]. Variations of CLDN1 and CLDN2 expression [8,11,26,39], as well as the reduction of occludin [10,40], have been reported as a key factor triggering alterations of intestinal permeability. To the best of our knowledge, to date there are no studies available showing the relationship between miR-195-5p and tight junctions’ regulation in IBD.

We found an increased expression of Cldn2 in the UC mice model and showed that miR-195-5p expression was negatively correlated with the Cldn2 expression levels in the same set of IECs samples.

According to our bioinformatic analysis, in human colonic epithelial cell lines, after raising the levels of intracellular miR-195-5p, the levels of Cldn2 mRNA and protein were reduced. This modulation, in turn, downregulated CLDN1 expression at a protein level, but not that of occludin.

Finally, we determined the clinical significance of miR-195-5p and associated TJ genes in UC patients, finding that miR-195-5p resulted downregulated in UC patients and CLDN1 and CLDN2 resulted in upregulation. A downregulation of miR-195-5p has been previously reported in colon cancer tissue as a regulator of the EMT and M2-like TAM polarization through the NOTCH2-GATA3/IL-4 axis [41].

Altogether, our results highlight that modulation of TJ proteins by miR-195-5p may cause epithelial barrier disruption in the colon of patients with IBD, which could partially explain the dysfunction characteristic of this disease.

Our work has a limitation. In fact, it is a preliminary study aimed to demonstrate the modulation of TJ by miR-195-5p. Further experiments are needed to confirm the effective involvement of this miRNA in the regulation of intestinal barrier function in IBD mice models.

In conclusion, in this study, we show that miR-195-5p regulates CLDN2 and also, indirectly, CLDN1 protein. Our findings could have therapeutic implications, since the intracellular increase of miR-195-5p reverses the higher levels of CLDN2 TJ typical of IBD. Therefore, the use of miR-195-5p could provide a useful new therapeutic approach to the disease.

## Data Availability

Microarray data are available under accession number GSE183896 at the Gene Expression Omnibus (http://www.ncbi.nlm.nih.gov/geo/, accessed on 25 February 2022).

## Supplementary Materials

The following supporting information can be downloaded at: https://www.mdpi.com/2227-9059/10/4/919/s1, Table S1: List of 18 miRNAs differently expressed in 4 ulcerative colitis mice models compared to 4 wild-type mice (FDR ≤ 0.05 and fold change threshold ≥2) using microarray technology; Table S2: miR-195a-5p predicted target; Table S3: Pathway analysis of 18 dysregulated miRNAs.

## Appendix A

Raw data of the independent experiments of Western blot.

## Appendix B

Single immunofluorescence images for each channel of the three cell lines in all conditions.

## References

[1] Zhang Y.-Z. (2014). Inflammatory bowel disease: Pathogenesis. World J. Gastroenterol..

[2] Ramos G.P., Papadakis K.A. (2019). Mechanisms of Disease: Inflammatory Bowel Diseases. Mayo Clin. Proc..

[3] Anderson J.M., Van Itallie C.M. (2009). Physiology and Function of the Tight Junction. Cold Spring Harb. Perspect. Biol..

[4] Landy J., Ronde E., English N., Clark S.K., Hart A.L., Knight S.C., Ciclitira P.J., Al-Hassi H.O. (2016). Tight junctions in inflammatory bowel diseases and inflammatory bowel disease associated colorectal cancer. World J. Gastroenterol..

[5] Krause G., Winkler L., Mueller S.L., Haseloff R.F., Piontek J., Blasig I.E. (2008). Structure and function of claudins. Biochim. Biophys. Acta Biomembr..

[6] Bücker R., Schumann M., Amasheh S., Schulzke J.-D. (2010). Claudins in Intestinal Function and Disease. Current Topics in Membranes.

[7] Blikslager A.T., Moeser A.J., Gookin J.L., Jones S.L., Odle J. (2007). Restoration of Barrier Function in Injured Intestinal Mucosa. Physiol. Rev..

[8] Zhu L., Han J., Li L., Wang Y., Li Y., Zhang S. (2019). Claudin Family Participates in the Pathogenesis of Inflammatory Bowel Diseases and Colitis-Associated Colorectal Cancer. Front. Immunol..

[9] Luettig J., Rosenthal R., Barmeyer C., Schulzke J. (2015). Claudin-2 as a mediator of leaky gut barrier during intestinal inflammation. Tissue Barriers.

[10] Weber C.R., Nalle S.C., Tretiakova M., Rubin D.T., Turner J.R. (2008). Claudin-1 and claudin-2 expression is elevated in inflammatory bowel disease and may contribute to early neoplastic transformation. Lab. Investig..

[11] Suzuki T., Yoshinaga N., Tanabe S. (2011). Interleukin-6 (IL-6) Regulates Claudin-2 Expression and Tight Junction Permeability in Intestinal Epithelium. J. Biol. Chem..

[12] Bartel D.P. (2004). MicroRNAs. Cell.

[13] Kalla R., Ventham N.T., Kennedy N.A., Quintana J.F., Nimmo E.R., Buck A.H., Satsangi J. (2015). MicroRNAs: New players in IBD. Gut.

[14] Wang C., Chen J. (2019). microRNAs as therapeutic targets in intestinal diseases. ExRNA.

[15] Dalal S.R., Kwon J.H. (2010). The Role of MicroRNA in Inflammatory Bowel Disease. Gastroenterol. Hepatol..

[16] Chapman C.G., Pekow J. (2015). The emerging role of miRNAs in inflammatory bowel disease: A review. Therap. Adv. Gastroenterol..

[17] Altaf-Ul-Amin M., Karim M.B., Hu P., ONO N., Kanaya S. (2020). Discovery of inflammatory bowel disease-associated miRNAs using a novel bipartite clustering approach. BMC Med. Genom..

[18] Sturn A., Quackenbush J., Trajanoski Z. (2002). Genesis: Cluster analysis of microarray data. Bioinformatics.

[19] Kozomara A., Griffiths-Jones S. (2014). miRBase: Annotating high confidence microRNAs using deep sequencing data. Nucleic Acids Res..

[20] Sticht C., De La Torre C., Parveen A., Gretz N. (2018). miRWalk: An online resource for prediction of microRNA binding sites. PLoS ONE.

[21] Agarwal V., Bell G.W., Nam J.-W., Bartel D.P. (2015). Predicting effective microRNA target sites in mammalian mRNAs. Elife.

[22] Karagkouni D., Paraskevopoulou M.D., Chatzopoulos S., Vlachos I.S., Tastsoglou S., Kanellos I., Papadimitriou D., Kavakiotis I., Maniou S., Skoufos G. (2018). DIANA-TarBase v8: A decade-long collection of experimentally supported miRNA–gene interactions. Nucleic Acids Res..

[23] Chen Y., Wang X. (2020). miRDB: An online database for prediction of functional microRNA targets. Nucleic Acids Res..

[24] Lu T.-P., Lee C.-Y., Tsai M.-H., Chiu Y.-C., Hsiao C.K., Lai L.-C., Chuang E.Y. (2012). miRSystem: An Integrated System for Characterizing Enriched Functions and Pathways of MicroRNA Targets. PLoS ONE.

[25] Vlachos I.S., Zagganas K., Paraskevopoulou M.D., Georgakilas G., Karagkouni D., Vergoulis T., Dalamagas T., Hatzigeorgiou A.G. (2015). DIANA-miRPath v3.0: Deciphering microRNA function with experimental support. Nucleic Acids Res..

[26] Amasheh S., Meiri N., Gitter A.H., Schöneberg T., Mankertz J., Schulzke J.D., Fromm M. (2002). Claudin-2 expression induces cation-selective channels in tight junctions of epithelial cells. J. Cell Sci..

[27] Förster C. (2008). Tight junctions and the modulation of barrier function in disease. Histochem. Cell Biol..

[28] Antoni L. (2014). Intestinal barrier in inflammatory bowel disease. World J. Gastroenterol..

[29] Okumura R., Takeda K. (2017). Roles of intestinal epithelial cells in the maintenance of gut homeostasis. Exp. Mol. Med..

[30] Bischoff S.C., Barbara G., Buurman W., Ockhuizen T., Schulzke J.-D., Serino M., Tilg H., Watson A., Wells J.M. (2014). Intestinal permeability—A new target for disease prevention and therapy. BMC Gastroenterol..

[31] Zhou H., Xiao J., Wu N., Liu C., Xu J., Liu F., Wu L. (2015). MicroRNA-223 Regulates the Differentiation and Function of Intestinal Dendritic Cells and Macrophages by Targeting C/EBPβ. Cell Rep..

[32] Shi Y., Dai S., Qiu C., Wang T., Zhou Y., Xue C., Yao J., Xu Y. (2020). MicroRNA-219a-5p suppresses intestinal inflammation through inhibiting Th1/Th17-mediated immune responses in inflammatory bowel disease. Mucosal Immunol..

[33] Galleggiante V., De Santis S., Liso M., Verna G., Sommella E., Mastronardi M., Campiglia P., Chieppa M., Serino G. (2019). Quercetin-Induced miR-369-3p Suppresses Chronic Inflammatory Response Targeting C/EBP-β. Mol. Nutr. Food Res..

[34] Scalavino V., Liso M., Serino G. (2020). Role of microRNAs in the Regulation of Dendritic Cell Generation and Function. Int. J. Mol. Sci..

[35] Scalavino V., Liso M., Cavalcanti E., Gigante I., Lippolis A., Mastronardi M., Chieppa M., Serino G. (2020). miR-369-3p modulates inducible nitric oxide synthase and is involved in regulation of chronic inflammatory response. Sci. Rep..

[36] Dai X., Chen X., Chen Q., Shi L., Liang H., Zhou Z., Liu Q., Pang W., Hou D., Wang C. (2015). MicroRNA-193a-3p Reduces Intestinal Inflammation in Response to Microbiota via Down-regulation of Colonic PepT1. J. Biol. Chem..

[37] Zhang L., Shen J., Cheng J., Fan X. (2015). MicroRNA-21 regulates intestinal epithelial tight junction permeability. Cell Biochem. Funct..

[38] Tian Y., Xu J., Li Y., Zhao R., Du S., Lv C., Wu W., Liu R., Sheng X., Song Y. (2019). MicroRNA-31 Reduces Inflammatory Signaling and Promotes Regeneration in Colon Epithelium, and Delivery of Mimics in Microspheres Reduces Colitis in Mice. Gastroenterology.

[39] Poritz L.S., Harris L.R., Kelly A.A., Koltun W.A. (2011). Increase in the Tight Junction Protein Claudin-1 in Intestinal Inflammation. Dig. Dis. Sci..

[40] Marchiando A.M., Shen L., Graham W.V., Weber C.R., Schwarz B.T., Austin J.R., Raleigh D.R., Guan Y., Watson A.J.M., Montrose M.H. (2010). Caveolin-1–dependent occludin endocytosis is required for TNF-induced tight junction regulation in vivo. J. Cell Biol..

[41] Lin X., Wang S., Sun M., Zhang C., Wei C., Yang C., Dou R., Liu Q., Xiong B. (2019). miR-195-5p/NOTCH2-mediated EMT modulates IL-4 secretion in colorectal cancer to affect M2-like TAM polarization. J. Hematol. Oncol..

